# How does the foraging behavior of large herbivores cause different associational plant defenses?

**DOI:** 10.1038/srep20561

**Published:** 2016-02-05

**Authors:** Yue Huang, Ling Wang, Deli Wang, De-Hui Zeng, Chen Liu

**Affiliations:** 1 Institute of Grassland Science, Northeast Normal University, Key Laboratory of Vegetation Ecology, Ministry of Education, 5268 Renmin St., Changchun 130024, P.R. China; 2 State Key Laboratory of Forest and Soil Ecology, Institute of Applied Ecology, Chinese Academy of Sciences, 72 Wenhua Road, Shenyang 110016, P.R. China; 3 Northwest Polytechnical University, Centre for Ecological and Environmental Sciences, Xi’an, 710072, P.R. China

## Abstract

The attractant-decoy hypothesis predicts that focal plants can defend against herbivory by neighboring with preferred plant species when herbivores make decisions at the plant species scale. The repellent-plant hypothesis assumes that focal plants will gain protection by associating with nonpreferred neighbors when herbivores are selective at the patch scale. However, herbivores usually make foraging decisions at these scales simultaneously. The net outcomes of the focal plant vulnerability could depend on the spatial scale at which the magnitude of selectivity by the herbivores is stronger. We quantified and compared the within- and between-patch overall selectivity index (OSI) of sheep to examine the relationships between associational plant effects and herbivore foraging selectivity. We found that the sheep OSI was stronger at the within- than the between-patch scale, but focal plant vulnerability followed both hypotheses. Focal plants defended herbivory with preferred neighbors when the OSI difference between the two scales was large. Focal plants gained protection with nonpreferred neighbors when the OSI difference was narrowed. Therefore, the difference in selectivity by the herbivores between the relevant scales results in different associational plant defenses. Our study suggests important implications for understanding plant-herbivore interactions and grassland management.

Plants are commonly reported to defend against herbivory by lowering herbivore foraging efficiency using their morphological and chemical characteristics^1^,^2^,^3^,^4^,^5^,^6^,^7^. However, an increasing amount of evidence demonstrates that the existence of neighboring plants can produce associational effects on the degree of protection experienced by focal plants^8^,^9^,^10^,^11^,^12^,^13^,^14^.

There are two main hypotheses that predict how neighbor plants help target species to defend against herbivory. One hypothesis posits that a plant that is susceptible to herbivory can gain protection when it is associated with a more preferred plant species in a high-quality patch, which is referred to as the attractant-decoy hypothesis because herbivore attention is diverted to the more preferred neighbors^15^,^16^. However, many studies show results consistent with the repellent-plant hypothesis, which supports the opposite prediction that a focal plant will escape animal attack when it is associated with a less preferred neighbor in a low-quality patch; this occurs because herbivores avoid visiting the bad patch and concentrate their foraging efforts on the more beneficial patches^17^,^18^.

These conflicting predictions from the two hypotheses have been attributed to the spatial scale of herbivore foraging decisions^15^,^19^. When herbivores are selective between patches and make foraging decisions mainly at the patch scale, target plants will escape foraging damage in the bad patch. Thus, the associational effects are consistent with the repellent-plant hypothesis^20^,^21^. Conversely, if the animals are selective within a patch and make foraging strategies at the plant species scale, the attractant-decoy hypothesis will account for the vulnerability of the focal plants^15^,^22^. However, large herbivores usually make foraging decisions at these spatial scales^23^,^24^,^25^,^26^,^27^,^28^ simultaneously, and decisions at one level can be modified by decisions at other levels^29^,^30^. Associational plant defenses may therefore result from the net foraging decisions that are made by the herbivores at these relevant spatial scales, relying particularly on the spatial scale at which the magnitude of selectivity by the herbivores is stronger. However, few studies have quantified herbivore foraging selectivity and directly compared the magnitude of selectivity at within- and between-patch scales to examine the associational plant effects.

Large herbivores have been reported to precisely discriminate among the different plant species and to make efficient judgments among different quality patches^31^,^32^,^33^,^34^. The trade-off of herbivore foraging selectivity at the within- and between-patch scales strongly depends on the quality contrast between the plant species or between the patches^35^,^36^,^37^,^38^,^39^,^40^. However, it remains uncertain how the trade-offs of herbivore foraging decisions between different quality plant species and patches regulate the associational plant effects. In this study, we established different quality contrasts between patches and between plant species within a patch for sheep to graze in to examine the relationships between the magnitude of foraging selectivity at within- and between-patch scales and the consequent associational plant effects. Specifically, we hypothesized the following (Fig. 1): (1) if the quality contrast is high between plant species and low between patches, the within-patch selectivity of sheep will be higher than the between-patch selectivity, focal plants will gain protection in the palatable patches, and the associational plant effects will be consistent with the attractant-decoy hypothesis (represented by the dash line ‘––’); (2) when the quality contrast between patches and between species is consistently high or low, foraging selectivity of sheep at within- and between-patch scales will be similar, and thus, there will be no obvious neighboring associational effects (represented by the solid line ‘—’); and (3) when the quality contrast is low between plant species and high between patches, and the between-patch selectivity will exceed the within-patch selectivity, focal plants will escape herbivore attack in the unpalatable patches, and the associational plant effects will switch and become consistent with the repellent-plant hypothesis (represented by the dot line ‘….’).

## Results

### The vulnerability of focal plants with different neighbors

**Figure 2:**
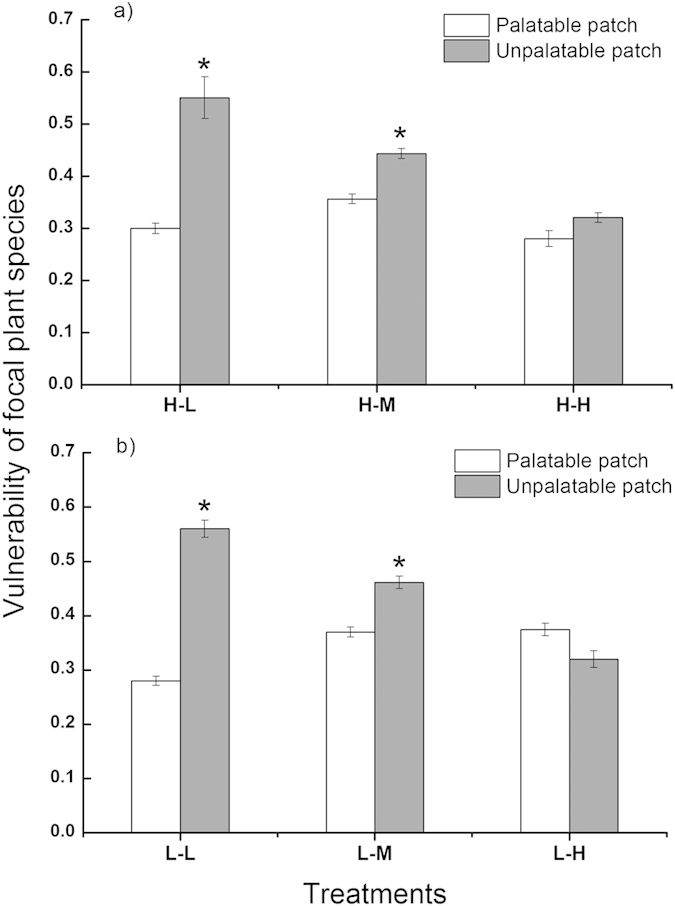
Effects of between-patch quality contrast on the vulnerability of focal plants to sheep grazing in the palatable and unpalatable patches in the high (**a**) and low (**b**) within-patch quality contrast treatments, respectively. H-L: high quality contrast within patches and low contrast between patches; H-M: high contrast within patches and medium contrast between patches; H-H: high contrast within and between patches. L-L: low contrast within and between patches; L-M: low contrast within patches and medium contrast between patches; L-H: low contrast within patches and high contrast between patches. The values are the means (±SE) for the five groups of three sheep measured four times within each treatment. The values with an asterisk are significantly different (*P* < 0.05).

Within- and between-patch quality contrast significantly affected the sheep consumption of focal plants associated with different quality neighbor species (Fig. 2). The vulnerability of the focal plant, *Kalimeris integrifolia*, was significantly lower when neighbored with *Chloris virgata* or *Lathyrus quinquenervius*, the preferred species, in the palatable patches than when neighbored with *Artemisia scoparia*, the nonpreferred species, in the unpalatable patches in the four treatments of low and medium between-patch quality contrast (treatments H-L, H-M, L-L and L-M) (*P* = 0.008, 0.006, 0.0001 and 0.003 for treatments H-L, H-M, L-L and L-M, respectively; Fig. 2a,b). These results were consistent with the predictions of the attractant-decoy hypothesis. However, nearly the same amounts of focal plants were consumed in both the bad and good patches when the between-patch contrast was high and the within-patch contrast was also high (treatment H-H) and where there was no obvious associational effect (*P* = 0.12, Fig. 2a). Furthermore, the vulnerability of *K. integrifolia* in the good patches showed a trend to overwhelm that in the bad patches, although this tendency was not statistically significant (*P* = 0.071, Fig. 2b) when the within-patch quality contrast was low and the between-patch contrast was high (treatment L-H). In such a case, associational plant effects switched to the direction predicted by the repellent-plant hypothesis.

### Sheep foraging decisions at within- and between-patch scales

**Figure 3:**
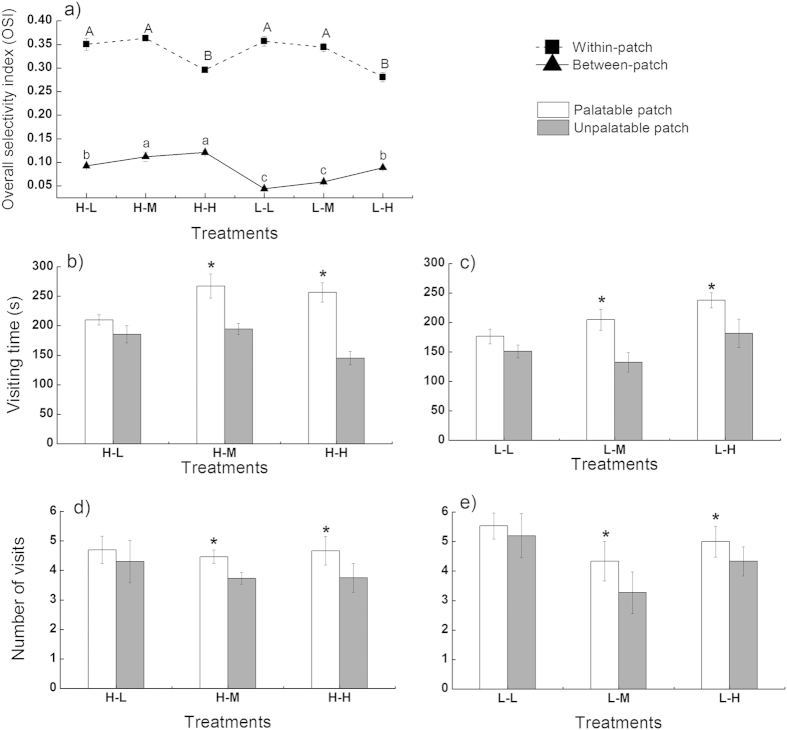
Sheep overall selectivity index (OSI) at the within- and between-patch scales as affected by the within- and between-patch quality contrast (**a**); sheep visiting time (**b,c**) and visiting numbers (**d,e**) for the palatable and unpalatable patches as affected by the within- and between-patch quality contrast. The between-patch OSI is calculated across all of the patches; the within-patch OSI is calculated across all of the species within a patch, and the values of all the patches are averaged. The values are the means (±SE) for the five groups of three sheep measured four times within each treatment. The values with different letters or asterisks are significantly different (*P* < 0.05).

Generally, the sheep overall selectivity index (OSI) was significantly and consistently higher within patches than between patches in all of the treatments (Fig. 3a, *P* < 0.0001). The increased between-patch quality contrast significantly increased the between-patch OSI and reduced the within-patch OSI (*P* < 0.05). The between-patch OSI was significantly lower when the between-patch contrast was low than when the between-patch contrast was medium or high. Additionally, the within-patch OSI was significantly lower when the between-patch contrast was high than when the between-patch contrast was medium or low. Furthermore, the within-patch quality contrast significantly decreased the between-patch OSI but did not affect the within-patch OSI (Fig. 3a).

The sheep visiting time and the number of visits to each patch were also affected by the within- and between-patch quality contrasts (Fig. 3b–e). Sheep took more time and made more visits to the palatable patches than to the unpalatable patches when the between-patch quality contrast was medium or high (*P* < 0.05, treatments L-M, L-H, H-M and H-H). However, no significant differences in visiting time or the number of visits between the good and bad patches were observed when the between-patch contrast was low (treatments H-L and L-L, *P* > 0.05). Specifically, sheep spent 1.8 times more time in the good patches than in the bad patches when both the within- and between-patch contrast were high, indicating that the sheep were more selective between patches (treatment H-H, *P* = 0.0063).

### The relationships between associational plant effects and sheep foraging selectivity

**Figure 4:**
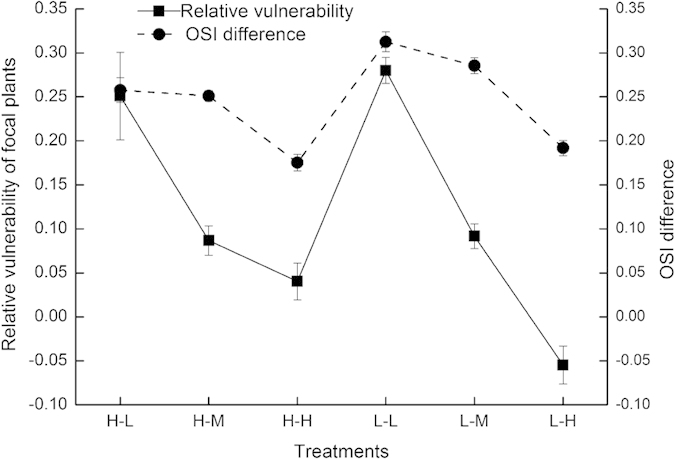
Relationships between the relative vulnerability of focal plants at two patches and the OSI difference at the within- and between-patch scales. The values are the means (±SE) for the five groups of three sheep measured four times within each treatment. The values with different letters are significantly different (*P* < 0.05).

There was a positive correlation between the sheep OSI difference of within- and between-patch scales and the relative vulnerability of the focal plants between the palatable and unpalatable patches (*R*^2^ = 0.593, *P* = 0.073) (Fig. 4). As the between-patch quality contrast increased, whenever the within-patch contrast was low or high, the relative vulnerability of the focal plants between the unpalatable and palatable patches decreased, and the within- and between-patch OSI differences were diminished. Focal plant vulnerability followed the attractant-decoy hypothesis when the relative vulnerability of the focal plants was positive (treatment L-L, L-M, H-L and H-M), showed no obvious associational plant effects when the relative vulnerability of the focal plants trended to zero (treatment H-H), and proceeded in the direction predicted by the repellent-plant hypothesis when the relative vulnerability of the focal plants was negative (treatment L-H).

## Discussion

Our study is among the first to provide empirical evidence that different associational plant effects result from the magnitude difference of within- and between-patch foraging selectivity by herbivores. Previous studies have shown that neighboring plant effects or the risk of focal plants to herbivory depend on the spatial scales at which herbivores make foraging decisions^18^,^22^,^41^. However, these studies overlooked the synchronicity and interactions of the foraging strategies at multiple spatial scales^26^,^29^ and failed to quantify and compare the foraging selectivity to predict the associational plant effects and the consequent vulnerability of focal plants in the highly heterogeneous grassland environments. By calculating the sheep OSI, our study demonstrated that sheep were more selective at the within- than between-patch scale, and the magnitude of within-patch selectivity was much higher than that of between-patch selectivity even if the quality contrast between the patches was very high. However, the difference of magnitude in the within- and between-patch selectivity can still lead to different associational plant effects, which was not consistent with previous studies and our hypotheses.

In our study, the focal plant, *K. integrifolia*, was less attacked by sheep when it was associated with preferred neighbors in the good patches than when it was associated with the nonpreferred species in the bad patches when the between-patch quality contrast was low (treatments H-L and L-L). This result is in accordance with the predictions of the attractant-decoy hypothesis. The sheep within-patch OSI was much higher than the between-patch OSI, which indicated that the sheep were more selective between plant species (Fig. 3a). Previous studies have also reported that some mammal herbivores (e.g., cattle, squirrels and deer) are highly selective at the within-patch scale and, consequently, cause focal plants to escape animal attack when neighboring with preferred species^15^,^22^,^42^. Herbivores can discriminate among different plant species and always preferentially choose the most nutritional food by using visual, olfactory or chemical cues^43^,^44^,^45^,^46^. Therefore, during our experiment, the sheep were first attracted by the neighbor plants (*C. virgata*) because of their higher palatability and, consequently, consumed less of the focal species in the good patches.

Focal plants still gained protection in the good patches (Fig. 2) when the between-patch contrast increased to medium (treatments H-M and L-M). However, the between-patch selectivity by the sheep was significantly improved, and the selectivity difference between the two scales became smaller because of the increased between-patch quality contrast (Figs 3a and 4). Therefore, the relative vulnerability of focal plants between the good and bad patches was greatly diminished. However, the consumption of focal plants at the good and bad patches was similar when the between-patch quality contrast was further increased (treatments H-H and L-H), which led to no obvious associational plant effects. Increasing the between-patch quality contrast can increase sheep selectivity at the between-patch scale. Sheep spent more time and made more visits to the good patches, which showed that the sheep could discriminate precisely between patches (Fig. 3b–e). Although the sheep between-patch selectivity was still lower than the within-patch selectivity, the difference of the magnitude in the within- and between-patch foraging selectivity was greatly narrowed (Figs 3a and 4). In treatment L-H, the vulnerability of the focal plants was even marginally higher at the good than at the bad patches (*P* = 0.071, Fig. 2b), and the associational plant effects proceeded in the direction predicted by the repellent-plant hypothesis. This low quality contrast between species and high contrast between patches can strengthen sheep selectivity at the between-patch scale^14^,^39^,^41^. Sheep frequently visited the good patches and spent more time feeding there (Fig. 3c,e). Therefore, the risk of the focal plants to be eaten by sheep increased in the good patches and decreased in the bad patches. Consequently, the focal plant vulnerability switched from the bad patches to the good patches.

The relationships of herbivore foraging selectivity and associational plant effects found in our study are markedly different from the relationships observed in previous experimental studies. Bergvall *et al.* (2006)^15^ found that fallow deer were selective at the within-patch scale and nonselective at the between-patch scale, and the focal plants followed the attractant-decoy hypothesis. Miller *et al.* (2007, 2009)^19^,^22^ also found that focal plant vulnerability followed the attractant-decoy hypothesis with red-bellied pademelons when the animals did not have the opportunity to make choices at the between-patch scale. However, when given the opportunity, the animals preferentially chose between patches, and the vulnerability of the focal plant was consistent with the repellent-plant hypothesis. However, neither of these studies directly quantified and compared foraging selectivity at within- and between-patch scales and only used herbivore behavioral variables (e.g., patch residence time or switching frequency among patches) to explain at which spatial scale the herbivores make foraging decisions. By calculating the herbivore OSI and comparing the selectivity intensity at within- and between-patch scales, we found that herbivores made foraging decisions simultaneously at within- and between-patch scales in our study. Focal plant vulnerability follows both the attractant-decoy hypothesis and the repellent-plant hypothesis, although herbivore foraging selectivity is stronger at the within- than the between-patch scale. The outcomes of a focal plant defense to herbivory associated with neighbor species results from the intensity difference of foraging selectivity at the two spatial scales.

Additionally, sheep had a much higher within- than between-patch foraging selectivity in our study, although we greatly reduced the within-patch quality contrast and increased the between-patch quality contrast. The average within-patch OSI was 0.332 ± 0.01, which was almost four times higher than the between-patch OSI (0.086 ± 0.01). This may have occurred because the nutrient requirement is the most important factor determining the food choice of sheep. In our previous studies, the sheep always consumed a large amount of the most preferred species, *L. quinquenervius* or *Medicago sativa,* prior to consuming other less preferred species^40^,^47^. Moreover, in an experiment testing how the species distribution pattern at the population level affected associational plant effects, we also found that the sheep had a very high within-patch foraging selectivity compared with the between-patch foraging selectivity^14^. However, there is still a lack of evidence on the food choices of other large mammal herbivores at the within- and between-patch scales and on how their foraging selectivity modifies associational plant effects under more complex and heterogeneous foraging environments. Hence, we advocate the use of the OSI index to quantify the magnitude of selectivity of other herbivores and to improve our understanding of the behavioral mechanisms by which focal plants defend the herbivory associated with neighbor species. Our findings bear important implications not only for understanding the complex plant-herbivore interaction patterns under heterogeneous environments but also for the management of native plant communities and particularly for the conservation of some rare and endangered plant species.

## Methods

### Ethics statement

The methods and the caring for sheep were carried out in accordance with the guidelines set by the Northeast Normal University. All experimental protocols were approved by the Institute of Grassland Science, Northeast Normal University of China.

### Plant species and experimental animals

This experiment was conducted at the Grassland Ecological Research Station of Northeast Normal University, Jilin Province, P.R. China (44°40′–44°44′N and 123°44′–123°47′E). We used sheep as our model mammalian herbivore. Fifteen 2-year-old male Northeast fine-wool sheep (body weight 34.5 ± 1.51 kg, mean ± SE) that were bred in Northeast China were used in this experiment.

We prepared four native plant species, which formed the main dietary components of the sheep in the meadow steppe. *C. virgata* is an annual grass with the highest palatability to sheep in July. *L. quinquenervius* is a legume with high protein content. *K. integrifolia* is our focal plant with an intermediate nutrient content and medium palatability to sheep. *A. scoparia* is a forb with a high content of secondary compounds, and to the sheep, it is the food of lowest palatability among the four plants. The relative preference of these plants by the sheep was determined in preliminary tests. Each sheep was given the above four plants and *Leymus chinensis* at the same time. Four hundred grams of each species was offered in a separate trough. Because *L. chinensis* is the dominant species in the steppe in China and is a highly unpalatable species to sheep^11^, we used it as our background species in the grazing plots. The preference indexes (expressed as percentages, dividing the intake of each food by the total intake of all five species) for *C. virgata, L. quinquenervius, K. integrifolia, A. scoparia* and *L. chinensis* were 41 ± 3.5%, 28 ± 3.1%, 18 ± 3.5%, 11 ± 2.3% and 2 ± 0.4%, respectively. We transplanted four *L. quinquenervius* seedlings, four *K. integrifolia* seedlings and four *A. scoparia* seedlings from adjacent grasslands into pots (20 cm in diameter, 15 cm high) by species in early June in 2010. *C. virgata* was transplanted in late May in 2011. All of the plants were watered twice a week. There were 900 pots of *K. integrifolia* and 500 pots each of *C. virgata, L. quinquenervius* and *A. scoparia* before the experiment started.

### Experimental treatments and design

**Figure 5:**
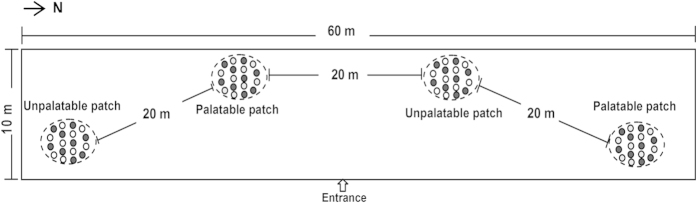
The experimental arena and an example of the treatment (H-M) layout are shown. The ellipses represent the patches and the inside spots represent the plants species: filled circle , focal plants; open circle , nonpreferred neighbor plants *Artemisia scoparia*; open circle with stippling , preferred neighbor plants *Chloris virgata*.

1. Quality contrast between plant species within a patch Two types of patches (palatable and unpalatable) were randomly distributed in one trial (Fig. 5). We defined a ‘patch’ as an area of approximately 6 m × 8 m, and each patch consisted of 18 pots equally spaced. Each distance between the centers of two adjacent pots within each patch was 1.5–2 m to provide enough space for the sheep to go back and forth between the pots. The palatable patches included focal plants that were neighbored with either *C. virgata* or *L. quinquenervius*, forming high or low quality contrast between the plant species, respectively. The unpalatable patches consisted of focal plants that were neighbored with the nonpreferred species *A. scoparia* in equal abundance.
2. Quality contrast between patches We changed the ratio between the neighbor and focal plants within the palatable patches to create high, medium or low quality contrast between the palatable and unpalatable patches, respectively. As a result, there were two levels of within-patch quality contrast crossed with three levels of between-patch quality contrast in a factorial treatment arrangement (see Table 1, Fig. 5 for details). The distance between the two adjacent patches was approximately 20 m. The quality contrast within a patch was less than that between any two patches^25^,^26^. The 15 sheep were randomly divided into five groups with three sheep in each group. All of the groups were allocated to the presentation of each food treatment, with one group at a time.

### Experimental procedure and sheep behavior observation

The foraging experiments were conducted in July and August 2011. The experimental field was a 10 × 60 m area with *L. chinensis* evenly distributed as the background plant species (Fig. 5). *L. chinensis* was the least preferred species to sheep because of its high acid detergent fiber in July (preference index was 2 ± 0.4%). Two identical palatable patches and two identical unpalatable patches were used in each trial. The sheep were trained daily for 15 days to walk into the forging area in groups and allowed to forage from the pots to become accustomed to consuming the plants in pots before the start of the experiment.

Each experimental trial was conducted from 4:00 to 8:00 am, which was the normal foraging time of sheep in this season. We measured the height of all of the plants in each pot before and after a trial to calculate how much of the plants had been eaten by the sheep. During the foraging process, three people stood outside the area and used digital voice recorders to record the foraging behavior of each sheep. After 7 minutes of grazing (almost ¾ of the pots had been visited and grazed), a trial was finished, and the sheep were removed to the holding pens. The five groups of sheep were tested only once a day. The experimental treatments were repeated four times. The pots that had been grazed on in any trial were moved back to the nursery and replaced with fresh ones prior to the following trial. During each trial, the positions of the four patches were exchanged randomly to preclude the possible effects of the sheep’s spatial memory on diet selection between trials. The sequence of the visits to the different patches and pots and the duration of each visit were recorded. A visit started when the sheep approached a patch and lowered its head into a pot and ended when it moved away from the patch.

To estimate the intake of each species in each patch, we cut 200 plants of each species at the ground level at the same time in the adjacent grasslands. All of the plant samples were dried at 65 °C for 48 h.

### Statistical analysis

The relationships between plant height and dry matter were examined and performed with PROC REG in SAS. Significant linear regressions (*P* < 0.05) existed between plant height and dry matter for all of the species (Table 2). Regression equations were used to calculate the dry matter of each plant offered to the animals before and after each trial, based on its height. The intake from each pot was then calculated as the difference in dry matter before and after each trial.

Computed foraging behavioral variables included the consumption of the focal plants in each patch, the visiting time (in seconds), the number of visits and the total food intake in each patch, the sheep OSI within and between patches and the OSI difference between the within- and between-patch scales. The OSI was indicated by the difference between the composition of the diet and the composition of available plants^48^ and was determined by the following equation^14^.


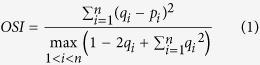


in which *p*_*i*_ is the mass proportion of plant species *i* (or patch *i*) consumed to total plant (patches) intake, *q*_*i*_ is the mass proportion of plant species *i* (or patch *i*) offered to total plants (patches) offered and *n* is the number of species (or patches) offered. The denominator is determined by the maximum value of the equation in the bracket calculating *q*_*i*_ with *n* different plant species or patches. OSI = 0 when the same proportion of each species (or patch) offered is consumed (i.e., completely nonselective), and OSI = 1 when only one species (or patch) is consumed (i.e., completely selective).

The vulnerability of the focal plants with different neighbors in the two patches was determined by the proportion of focal plants in the diet divided by the proportion of focal plants in the supply. The relative vulnerability of focal plants with different neighbors was obtained by the vulnerability of focal plants with preferred neighbors minus that with nonpreferred neighbors.

The vulnerability of the focal plants, visiting time, visiting numbers and within- and between-patch OSI between the palatable and unpalatable patches in each treatment were compared using a *T* test. The within- and between-patch OSI among the treatments were compared using a two-way ANOVA. The ANOVA model contained the quality contrast treatment as a fixed effect and the individual groups of sheep as blocks. The groups of three sheep were the units of replication. Behavioral data of the individual sheep were averaged for each group. Furthermore, we examined the relationships between the relative vulnerability of the focal plants and the OSI difference of within- and between-patch scales with Pearson’s correlation coefficient (n = 6). All of the statistical analyses were performed using the SAS 9.12 statistical package^49^. Assumptions of normality and heteroscedasticity were tested prior to the analyses. The significance level was set at *α* = 0.05.

## Additional Information

**How to cite this article**: Huang, Y. *et al.* How does the foraging behavior of large herbivores cause different associational plant defenses? *Sci. Rep.* **6**, 20561; doi: 10.1038/srep20561 (2016).

## Figures and Tables

**Figure 1:**
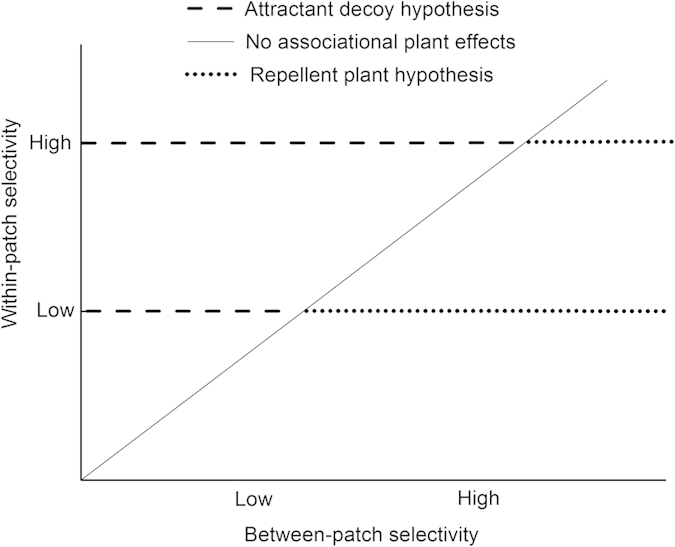
A hypothetical framework for the dynamics of associational plant effects as affected by the relative magnitude of within- and between-patch foraging selectivity by large herbivores . ‘––’ represents the attractant-decoy hypothesis because the within-patch selectivity is higher than that between patches. ‘—’ represents no associational plant effects because the within-patch selectivity becomes equal to between-patch selectivity. ‘.......’ represents the repellent-plant hypothesis because the within-patch selectivity becomes lower than that between patches.

**Table 1:** Experimental design and the numbers of pots of each species within each patch in each treatment.

**Treatments**	**Palatable patch**	**Unpalatable patch**
**H-L**	12 *K. integrifolia* + 6 *C. virgata*	9 *K. integrifolia* + 9 *A. scoparia*
**H-M**	9 *K. integrifolia* + 9 *C. virgata*	9 *K. integrifolia* + 9 *A. scoparia*
**H-H**	6 *K. integrifolia* + 12 *C. virgata*	9 *K. integrifolia* + 9 *A. scoparia*
**L-L**	12 *K. integrifolia* + 6 *L. quinquenervius*	9 *K. integrifolia* + 9 *A. scoparia*
**L-M**	9 *K. integrifolia* + 9 *L. quinquenervius*	9 *K. integrifolia* + 9 *A. scoparia*
**L-H**	6 *K. integrifolia* + 12 *L. quinquenervius*	9 *K. integrifolia* + 9 *A. scoparia*

Figures in the table indicate the number of pots of the species that follow. H-L: high quality contrast within patches and low contrast between patches; H-M: high contrast within patches and medium contrast between patches; H-H: high contrast within and between patches. L-L: low contrast within and between patches; L-M: low contrast within patches and medium contrast between patches; L-H: low contrast within patches and high contrast between patches. The high within-patch quality contrast treatments (H-L, H-M and H-H) were created by neighboring *C. virgata* with the focal plant *K. integrifolia*, and the low within-patch treatments (L-L, L-M and L-H) were created by neighboring *L. quinquenervius* with *K. integrifolia.* The between-patch quality contrast was made by changing the ratio between the neighbor and focal plants in the palatable patches and generating high, medium or low contrast levels to the fixed unpalatable patches.

**Table 2:** Regression equations of biomasses of the plant species in relation to their mean heights in the experiments.

**Species**	**Mean height ± SE (cm)**	**Mean biomass ± SE (g)**	***r***^***2***^	***y***
*Chloris virgata*	21.43 ± 0.56	0.32 ± 1.28	0.64	0.015 ×−1.73
*Lathyrus quinquenervius*	26.73 ± 0.41	0.82 ± 0.03	0.60	0.052 ×−0.59
*Kalimeris integrifolia*	24.04 ± 0.97	0.43 ± 0.03	0.79	0.026 ×−0.21
*Artemisia scoparia*	47.10 ± 0.70	2.42 ± 0.12	0.52	0.119 ×−3.23
*Leymus chinensis*	47.24 ± 0.84	0.82 ± 0.03	0.69	0.030 ×−0.63

‘x’ is the independent variable of the species’ height, and ‘y’ is the dependent variable of the species’ biomass.
